# Coccidioidomycosis—A Fungal Disease of the Americas

**DOI:** 10.1371/journal.pmed.0020002

**Published:** 2005-01-25

**Authors:** Richard F Hector, Rafael Laniado-Laborin

## Abstract

Coccidioidomycosis was first recognized as a serious disease over 100 years ago, but the disease remains an enigma and often goes undiagnosed, even in endemic areas

It has been more than a century since coccidioidomycosis was first recognized as a serious disease, and its etiology and epidemiology have been well documented. But the disease remains an enigma to many, and it often goes undiagnosed, even in endemic areas. As management of this chronic disease remains problematic, new preventive or therapeutic options are needed.

## Etiology and Epidemiology

**Figure 1:**
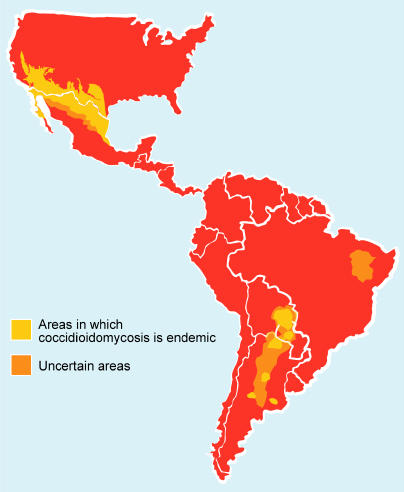
Geographic Distribution of Coccidioidomycosis (Illustration: Margaret Shear)

Coccidioidomycosis is a fungal disease found only in the Western Hemisphere. It is caused by two nearly identical species, Coccidioides immitis and C. posadasii, generically referred to as the “Californian” and “non-Californian” species respectively [1]. The fungus grows in a mycelial phase (see Box 1) in the soil within a geographically delineated area of the United States known as the Lower Sonoran Life Zone [2]. This semiarid zone encompasses the southern parts of Texas, Arizona, New Mexico, and much of central and southern California (Figure 1).

**Box 1. Glossary**

**Mycelial phase**—The growth form in the soil, composed of filamentous hyphae and reproductive spores called arthroconidia.

**Arthroconidia**—Reproductive spores, highly resistant to dessication, which are the infectious particles inhaled by man and animals.

**Spherules**—The parasitic phase of this dimorphic fungus; spherules are round cells of 30–100 µM or more that reproduce the progeny endospores.

**Endospores**—The progeny units of the parasitic phase, derived from spherules.

Endemic regions for coccidioidomycosis have long been identified in semiarid areas in Mexico [3], and smaller endemic foci have been described in areas of Central and South America [4,5]. More recently, Brazil has also been found to contain endemic areas in the semiarid northeastern states of the country [6]. The climatic conditions and flora of these states are similar to those in endemic regions in North, Central, and South America. In Latin America, Mexico has the largest number of reported cases, with the prevalence of infection in northern Mexico reported to be between 10%–40% [7,8]. C. posadasii is thought to be the predominant species in Mexico [3].

**Figure 2:**
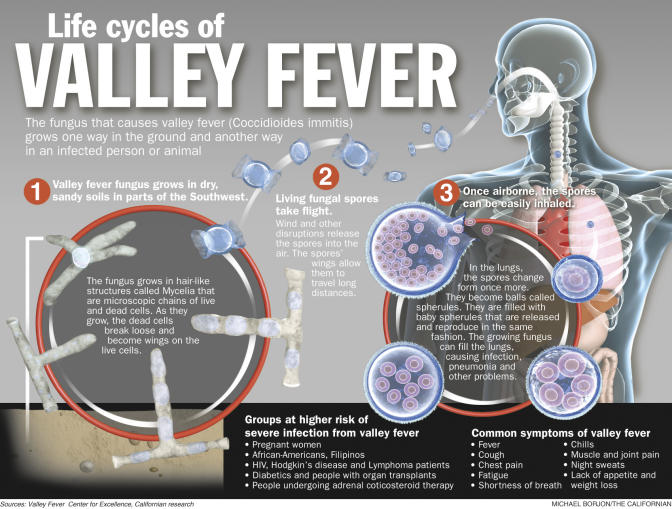
Life Cycle of Coccidioides immitis (Illustration: Michael Borjon/*The Bakersfield Californian*)

As the soil dries or nutrients become limiting, the fungus reproduces asexually by disarticulating the hyphae into small, environmentally-resistant arthroconidia (reproductive spores) (Figure 2). These are easily aerosolized when the soil is disturbed by wind or human activities. Consequently, it is the inhalation of the dust-borne arthroconidia that leads to infection by this pathogenic fungus in both humans and domestic or wild mammals. Upon inhalation, the fungus converts to a unique life cycle of alternating spherules and progeny endospores, which comprises the parasitic phase of this dimorphic fungus (Figure 2) [9]. Mycelial elements are only occasionally found in diseased tissue [10]. Coccidioidomycosis is not contagious; reports of human-to-human spread are extremely rare. Hence, primary exposure to contaminated dust is the sole risk factor for the acquisition of this disease.

It is estimated that upwards of 100,000 primary coccidioidal infections occur in humans each year in the endemic areas of the United States [11]. In recent years, the incidence of the disease has increased in California and Arizona, which may be partially due to the rapid immigration of previously unexposed persons from states outside the endemic areas (in other words, the pool of susceptible people has increased) [12]. In the United States, diagnosis in patients who have symptoms is established by serodiagnosis in conjunction with patient history. In previous decades, a coccidioidal skin test antigen was a useful adjunct in the diagnosis, but it became unavailable in the 1980s [13]. The incidence of primary pulmonary disease outside the United States is not established; most reports are limited to disseminated or unusual cases [14]. Diagnosis in Latin America is usually based on microbiologic findings, as serology is not always available [14].

## Clinical Features

In their pioneering epidemiologic studies, Smith and colleagues found that about 60% of exposures to the fungus result in asymptomatic infection [15]. In the 40% of patients who have symptomatic disease, there are protean manifestations. These range from a primary, or benign, pulmonary infection (commonly known as “Valley Fever”) to a progressive pulmonary or extrapulmonary disease involving the skin, bones and/or joints, the central nervous system, and other organ systems. Fortunately, most patients with primary disease recover spontaneously and retain lifelong immunity to exogenous reinfection. Chronic and disseminated disease is estimated to occur in up to 5% of infected individuals, with comparatively more cases occurring in older individuals and in males [12]. The most dangerous form of the disease is meningeal infection, which occurs in about 0.15%–0.75% of extrapulmonary coccidioidomycosis cases and requires treatment for life [16].

In regions where tuberculosis rates are high, the two diseases may occur together. Tuberculosis and coccidioidomycosis share common epidemiological, clinical, radiographic, and even histopathological features, making a correct diagnosis extremely difficult in cases where both diseases coexist. In areas where both diseases are endemic, the pertinent studies for diagnosing both conditions should be performed in every patient with compatible clinical features. The diagnosis of one of them does not exclude the possible existence of the other [17].

## Treatment

Historically, patients with the primary respiratory form of the disease were not treated because the vast majority recovered on their own. Instead, such patients were given supportive care and were monitored, often with radiographs, until the disease resolved. In recent years, however, an increasing number of physicians are prescribing azole antifungals in cases of primary disease, both because drugs like fluconazole have a good safety record, and because there is a perception that treatment may prevent progression to more serious forms of the disease. This latter presumption, however, is not supported by controlled trial data.

All cases of chronic or disseminated disease call for antifungal therapy, but the choice of drugs, route, and duration of therapy is highly dependent on the form of the disease, the severity and site(s) of infection, and the immune status of the patient. Galgiani and colleagues have published clinical practice guidelines on the choice of drug and duration of therapy for a given form of the disease [18].

There are only two classes of antifungal therapy routinely used for treatment of coccidioidomycosis. The first class is the polyenes, with amphotericin B desoxycholate and the newer lipid formulations used for the more serious forms of disease. The second class is the azoles, with ketoconazole, fluconazole, itraconazole, and the newer analogue voriconazole as available options. Voriconazole, in particular, is being used more and more often in life-threatening mycoses, and was found to be better than amphotericin B in the primary therapy of invasive aspergillosis [19]. According to available reports, treatment in Latin America usually consists of one of the azoles (fluconazole or itraconazole) and/or amphotericin B desoxycholate; lipid formulations are too costly to be accessible[20].

Treatment of the more serious or aggressive forms of the disease is typically of long duration and often results in less than complete resolution of disease; relapse is common [21]. Unfortunately, information on the treatment of coccidioidomycosis is limited, due to the small numbers of controlled trials performed for what is perceived to be a niche market. Clearly, newer, more powerful drugs are needed.

In addition to drugs, surgery is sometimes indicated to remove focalized infections, such as pulmonary cavities, or to debride osseous forms of the disease [22].

## Immunology and the Basis for a Vaccine

Acquired resistance to coccidioidomycosis strongly correlates with the development of a delayed-type hypersensitivity skin test response to coccidioidal antigens [23] and the production of T-helper-1 (Th1)-associated cytokines to coccidioidal antigens, such as interferon-gamma (IFN-γ) and Interleukin-2 (IL-2) [24]. Humoral immunity plays no known role in overcoming infection.

Although all humans are equally susceptible to initial infection, there is evidence of genetic predisposition to dissemination, independent of socioeconomic or environmental factors, particularly among African-Americans and Filipinos [25]. Pregnancy is also a risk factor.

In cases of marked immunosuppression, either in advanced AIDS or other forms of depressed cellular immunity, the management of coccidioidomycosis is particularly challenging and requires aggressive treatment [26].

As previously mentioned, recovery from disease confers lifelong immunity to reinfection, and is a rationale for the development and implementation of a vaccine for the prevention of symptomatic or serious forms of the disease. The combination of increasing incidence of disease, a growing population in the endemic area, and the lack of a highly effective drug treatment justifies efforts to prevent (rather than treat) this disease. To that end, a university-based consortium, the Valley Fever Vaccine Project (www.valleyfever.com), has identified and cloned immunogenic proteins that have proven effective in the prevention of deaths and fungal burdens in mouse models of coccidioidomycosis. This suggests that a vaccine for use in humans could be created [27]. A candidate vaccine comprised of a fusion protein based on two antigens has been selected and is currently in pharmaceutical development under the sponsorship of this project, with the goal of evaluating the safety and immunogenicity in humans.

## Conclusion

Although the vast majority of infected individuals emerge from coccidioidomycosis without complications, an unlucky minority are faced with a debilitating disease that lacks adequate drug options for rapid and completely effective treatment. In the absence of newer therapeutics, discoveries that lead to immunologic intervention [28] or prevention by vaccines may ultimately bring a measure of relief.
